# Macular hole following scleral buckling for rhegmatogenous retinal detachment: a case series

**DOI:** 10.1186/s12886-024-03324-w

**Published:** 2024-02-13

**Authors:** Fangyu Wang, Zhongqiao Zhu, Hong Yan, Yao Yang, Laxiao Niu, Jing Liu

**Affiliations:** 1 Shaanxi Eye Hospital, Xi’an People’s Hospital (Xi’an Fourth Hospital), No. 21 Jiefang Road, Xincheng District, 710004 Xi’an, Shaanxi China; 2 State Key Laboratory of Ophthalmology, Zhongshan Ophthalmic Center, Sun Yat-sen University, No. 54 Xianlienan Road, Yuexiu District, 510060 Guangzhou, Guangdong China

**Keywords:** Macular hole, Scleral buckling, Rhegmatogenous retinal detachment, Case series, Clinical study

## Abstract

**Background:**

Macular hole (MH) development following scleral buckling (SB) surgery for rhegmatogenous retinal detachment (RRD) repair is rare. This study presents both full-thickness MH (FTMH) and lamellar MH (LMH) cases following SB for the treatment of RRD.

**Methods:**

Clinical records of patients undergoing SB surgery for treatment of RRD at the Xi’an People’s Hospital (Xi’an Fourth Hospital) from January 2016 to December 2021 were reviewed, and cases with postoperative MH were selected. Clinical features and follow-up data were summarised, and possible causes were analysed.

**Results:**

Among 483 identified cases (483 eyes), four eyes (three male patients, one female patient) had postoperative MH, with prevalence, mean age, and mean axial length of 0.83%, 43.5 ± 10.66 years, and 29.13 ± 3.80 mm, respectively. All patients did not undergo subretinal fluid (SRF) drainage. The mean time for detecting MH was 26 ± 15.5 days postoperatively. Macula-off RRD with high myopia and FTMH combined with retinal re-detachment were diagnosed in three patients. One patient had macula-on RRD with outer LMH. The average follow-up duration was 7.25 ± 1.5 months. The FTMH closed successfully after reoperation, while the outer LMH closed without intervention. Visual acuity insignificantly improved or slightly decreased in all patients.

**Conclusions:**

Patients with high myopia combined with macula-off RRD might be more susceptible to FTMH, causing MH related retinal detachment. Additionally, LMH following SB was noted in patients with macula-on RRD. Therefore, we should raise awareness of MH following SB for RRD repair.

## Background

Macular hole (MH), first described by Knapp in 1869 [1], is a common disease that involves partial or complete impairment of the foveal tissue (from the retinal internal limiting membrane to the photoreceptor). The aetiology of MH could be either idiopathic or secondary. In contrast to idiopathic MH, secondary MH is caused by several factors, such as blunt trauma, uveitis, macular schisis, carcinoma, and surgery [2, 3]. Surgical repair for rhegmatogenous retinal detachment (RRD) can cause full-thickness MHs (FTMHs), which are even more common after vitrectomy [4–6]. However, MH following scleral buckling (SB) for the treatment of RRD is relatively rare [7]. To our knowledge, no case of MH following SB for RRD treatment has been previously reported in Chinese patients.

The distinction between MH and lamellar MH (LMH) is based on the degree of macular neuroepithelial tissue loss. Previous reports on MH following SB for RRD treatment were limited to FTMH, with no reported cases of LMH. We collected data from hospitalised patients who underwent SB for RRD treatment at Xi’an People’s Hospital (Xi’an Fourth Hospital) from January 2016 to December 2021. Four cases of postoperative MH following SB therapy, including both FTMH and LMH, were evaluated. In this article, we describe the clinical characteristics and prognosis of the four identified cases to supplement the available information for the comprehensive understanding of this rare postoperative complication and thus aid clinicians in making more rational and informed clinical decisions.

## Methods

This retrospective study was conducted in accordance with the ethical standards of the Declaration of Helsinki established in 1964 for research involving humans and was approved by the Medical Ethics Committee of Xi’an People’s Hospital (Xi’an Fourth Hospital). We reviewed clinical records of all patients who underwent SB for the treatment of RRD at Xi’an People’s Hospital (Xi’an Fourth Hospital) from January 2016 to December 2021 and studied cases that developed new MH lesions following surgery. The patients underwent a series of examinations, including best corrected visual acuity (BCVA) using the international standard visual acuity chart [8, 9], intraocular pressure (IOP) based on the readings from a non-contact tonometer (TX-20, Canon, Tokyo, Japan), slit-lamp test (YZ5X, 66 Vision-Tech, Suzhou, China), ophthalmoscope examination (YZ6H, 66 Vision-Tech), B-scan ultrasonography (Aviso, Quantel Medical, Cournon-d’Auvergne, France). B-scan ultrasound was performed on the patient in a supine position with closed eyelids, and appropriate amount of coupling agent was applied to the eyelids for measurement using B1-10 MHz probe (no contact with the cornea and without scleral shell). In addition, ultra-widefield retinal imaging (Daytona (P200T), Optos plc, Dunfermline, UK), and optical coherence tomography (OCT) (Spetralis OCT, Heidelberg Engineering, Heidelberg, Germany) were performed. Complete preoperative systemic examination was performed to exclude surgical contraindications. The indications of SB for RRD were as follows: the transparency of the lens and vitreous not affecting fundus observation; presence of peripheral retinal breaks; no strong vitreoretinal traction around retinal breaks; exclusion of MH following careful physical and OCT examination; lack of complex retinal detachment, such as giant retinal tears, proliferative vitreoretinopathy, and retinal dialysis; and patient’s full understanding of the possibility of reoperation. The operation was performed by two experienced vitreoretinal surgeons (ZZ and JL). The surgery was performed as follows. After disinfection and draping under local or general anaesthesia, the palpebral fissure was widely opened with an eye speculum and the bulbar conjunctiva was opened 360° along the limbus. Following this, the rectus muscle was suspended and the breaks and degenerating area were located under a binocular indirect ophthalmoscope (as the patient’s pupil was maximally dilated preoperatively) and were marked externally on the sclera. For single SB, a silicon sponge or band of appropriate size after designing was sutured to the scleral surface corresponding to the breaks. For cases combined with encircling, the encircling band was located at the equator and, according to the number and location of the breaks, a suitably sized silicon sponge or band was selected to be sutured to the scleral surface. An indirect ophthalmoscope was used to observe whether the scleral buckle ridge was raised and whether the relationship between the breaks and ridge was good. Appropriate adjustments were made if necessary, and the scleral buckle/encircling band was fixed. If there was a large quantity of subretinal fluid intraoperatively, the fluid was drained by puncturing the scleral surface corresponding to the most highly detached area before fixing the SB and gently pressing the eyeball with a cotton swab to assist the drainage of the subretinal fluid as much as possible. When no fluid drainage was performed, anterior chamber puncture was performed to release an appropriate amount of fluid to appropriately reduce the IOP before fixing the scleral buckle. Subsequently, the IOP was monitored. When the IOP was too low, an appropriate amount of disinfectant air was injected into the vitreous cavity; when the IOP was too high, an appropriate amount of aqueous humour was released. Furthermore, the central retinal artery was examined for patency. Finally, the Tenon capsule and conjunctiva were sutured. Patients were followed up for at least 6 months postoperatively. Data are expressed as mean ± standard deviation.

## Results

### Demographic features of all patients who underwent SB for RRD

Between January 2016 and December 2021, we assessed 483 patients (483 eyes) who underwent SB for the treatment of RRD. The demographic features of these cases are presented in Table 1. The average age of the patients was 50.2 ± 4.5 years, and 55.9% of the patients were male. More than half of the cases involved the right eye (59.8%), with one-third of RRD cases involving the macula (33.5%). Additionally, approximately 40% and > 50% of the cases of SB included a simultaneous encircling band (41.6%) and underwent subretinal fluid drainage (56.1%), respectively. Only four cases developed MH following the surgery, accounting for a prevalence of approximately 0.83%. We analysed the clinical data of these four cases to better understand this postoperative complication.

**Table 1:** Demographic characteristics of the study population (483 patients; 483 eyes)

Characteristics	Value
Age(years)	50.2 ± 4.5
Sex (male)	270 (55.9)
Eye (right)	289 (59.8)
Macular-off RRD	162 (33.5)
SB with encircling band	201 (41.6)
SF drainage	271 (56.1)
Secondary MH after SB	4 (0.8)

Values are presented as mean ± standard deviation or n (%)

MH: macular hole; RRD: rhegmatogenous retinal detachment; SB: scleral buckling; SF: subretinal fluid

### Characteristics of the four cases of MH following SB therapy for RRD

The baseline data of these four cases (three right eyes and one left eye) are presented in Table 2. The four cases involved three male patients and one female patient. The average age was 43.5 ± 10.66 years. Additionally, Cases 1, 2, and 3 had a 10-, 10-, and 24-year history of high myopia, respectively, while Case 4 had no such history. Only Case 1 had a history of an ocular disease; specifically, amblyopia diagnosed since childhood. The patients had RRD associated with a chief complaint of an average of 38.25 ± 54.96 days. Excluding the stated abnormalities, the patients were otherwise considered healthy.

**Table 2:** Baseline data of the four cases

	Patient 1	Patient 2	Patient 3	Patient 4
Sex	Male	Male	Male	Female
Age (years)	43	56	45	30
Eye laterality	Left	Right	Right	Right
Presence of high myopia (duration, years)	Yes (10)	Yes (10)	Yes (24)	No
Other ocular diseases	Amblyopia	No	No	No
General diseases	No	No	No	No
Course of RRD (days)	5	7	21	120

RRD: rhegmatogenous retinal detachment

The clinical characteristics of these cases are presented in Table 3. The average axial length was 29.13 ± 3.80 mm. Cases 1–3 had a long history of high myopia, and their axial length exceeded 26 mm. The axial length of Case 4 was normal. Phakic eye was present in all cases; posterior vitreous detachment (PVD) was present in Cases 1–3, but not in Case 4. Weiss rings were observed in Cases 1–3, but no obvious VMT was observed on OCT. Neither Weiss rings nor VMT were observed in Case 4. The range of retinal detachment and details of the retinal break of the four patients are described in Table 3. The retinal detachment of Cases 1–3 all involved the macula; therefore, all these patients had poor preoperative BCVA results. However, Case 4 did not have macular lesions and had good preoperative BCVA results. Since Case 4 had a long course of RRD, the IOP results were below normal. All four cases underwent SB surgery, encircling band, and subretinal fluid drainage and subsequently developed retinal reattachment postoperatively. After the operation, the patients underwent retinal photocoagulation to block the holes. Cases 2 and 4 had little residual subretinal fluid (the OCT images showed shallow subretinal fluid, while all retinal breaks were closed and the retina appeared attached under ophthalmoscopy) postoperatively, then disappeared at approximately 1 month and 3 weeks, respectively.

**Table 3:** Clinical characteristics of the four cases

	Patient 1	Patient 2	Patient 3	Patient 4
Axial length (mm)	33.8	29.2	29.0	24.5
Len status	Phakic	Phakic	Phakic	Phakic
PVD (Weiss ring/ obvious VMT on OCT)	Yes (Yes/ No)	Yes (Yes/ No)	Yes (Yes/ No)	No (No /No)
Retinal break	One 1/2 DD and one 1/3 DD oval holes at 12 o’clock	Three small round holes at 7 o’clock	One 2.5 DD horseshoe hole from 9 to 10 o’clock	Two small round holes at 12 and 1 o’clock
Range of RRD	9–3 o’clock	6–10 o’clock	3–12 o’clock	9–2 o’clock
Macula on/off	Off	Off	Off	On
Preoperative BCVA	0.02	CF/20 cm	HM/10 cm	0.8
Preoperative IOP (mmHg)	12.9	12.6	14.2	8
Major surgery performed	SB + EB + DSF	SB + EB + DSF	SB + EB + DSF	SB + EB + DSF
Retinal reattachment	Yes	Yes	Yes	Yes
Postoperative laser	Yes	Yes	Yes	Yes
Little residual SF	No	Yes	No	Yes

BCVA: best corrected visual acuity; CF: counting finger; DD: disc diameter; DSF: drainage of subretinal fluid; EB: encircling band; HM: hand movement; IOP: intraocular pressure; OCT: optical coherence tomography; PVD: posterior vitreous detachment; RRD: rhegmatogenous retinal detachment; SB: scleral buckling; SF: subretinal fluid; VMT: vitreomacular traction

### Follow-up information of the four cases

The follow-up information is presented in Table 4. After the SB operation, all patients were followed up for an average duration of 7.75 ± 1.26 months. The mean time for detection of postoperative MH development was 26 ± 15.5 days. Weiss rings were observed postoperatively in Cases 1–3, but no obvious VMT was observed on OCT. VMT was observed in Case 4, but without a Weiss ring. Cases 1–3 had signs of FTMH, whereas Case 4 had signs of outer LMH. The average size of MH for Cases 1–3 was 371 ± 139.82 μm, and all three cases had MH retinal detachment (MHRD) affecting the inferior quadrant. Owing to the MHRD, the patients underwent reoperation. Cases 1 and 3 underwent pars plana vitrectomy (PPV), internal limiting membrane peeling (ILMP), and silicon oil tamponade (SOT); Case 2 underwent PPV and air tamponade. In Cases 1 and 3, owing to the large range of retinal detachment (3–9 o’clock) and patients indicating reoperation to be acceptable, silicone oil tamponade was selected. In Case 2, the retinal detachment was observed only in a small range (7–9 o’clock), and the patient did not consider reoperation and had a family history of glaucoma; thus air tamponade was chosen. All MH lesions closed after the operations. Case 4 did not have FTMH or MHRD; thus, only regular follow ups were conducted. The last recorded IOP for all patients was normal.

**Table 4:** Follow-up data of the four cases

	Patient 1	Patient 2	Patient 3	Patient 4
Follow-up duration (months)	9	6	8	6
MH appearance time (days)	17	46	11	30
PVD (Weiss ring/ obvious VMT on OCT)	Yes (Yes/ No)	Yes (Yes/ No)	Yes (Yes/ No)	No (No /Yes)
Morphology of MH	Full thickness	Full thickness	Full thickness	Lamellar
MH size (µm)	226	382	505	-
MHRD	Yes	Yes	Yes	No
Range of MHRD	3–9 o’clock	7–9 o’clock	3–9 o’clock	None
Reoperation	PPV + ILMP + SOT	PPV + AT	PPV + ILMP + SOT	None
MH closed after operation	Yes	Yes	Yes	-
Retinal reattachment	Yes	Yes	Yes	-
Last BCVA	CF/10 cm	HM/BE	CF/BE	1.0
Last IOP (mmHg)	17.5	11.2	18.6	12.5

AT: air tamponade; BCVA: best corrected visual acuity; BE: before eye; CF: counting fingers; HM: hand movement; ILMP: internal limiting membrane peeling; IOP: intraocular pressure; MH: macular hole; MHRD: macular hole retinal detachment; OCT: optical coherence tomography; PPV: pars plana vitrectomy; PVD: posterior vitreous detachment; SOT: silicon oil tamponade; VMT: vitreomacular traction

### Imaging data of the four cases

**Fig. 1:**
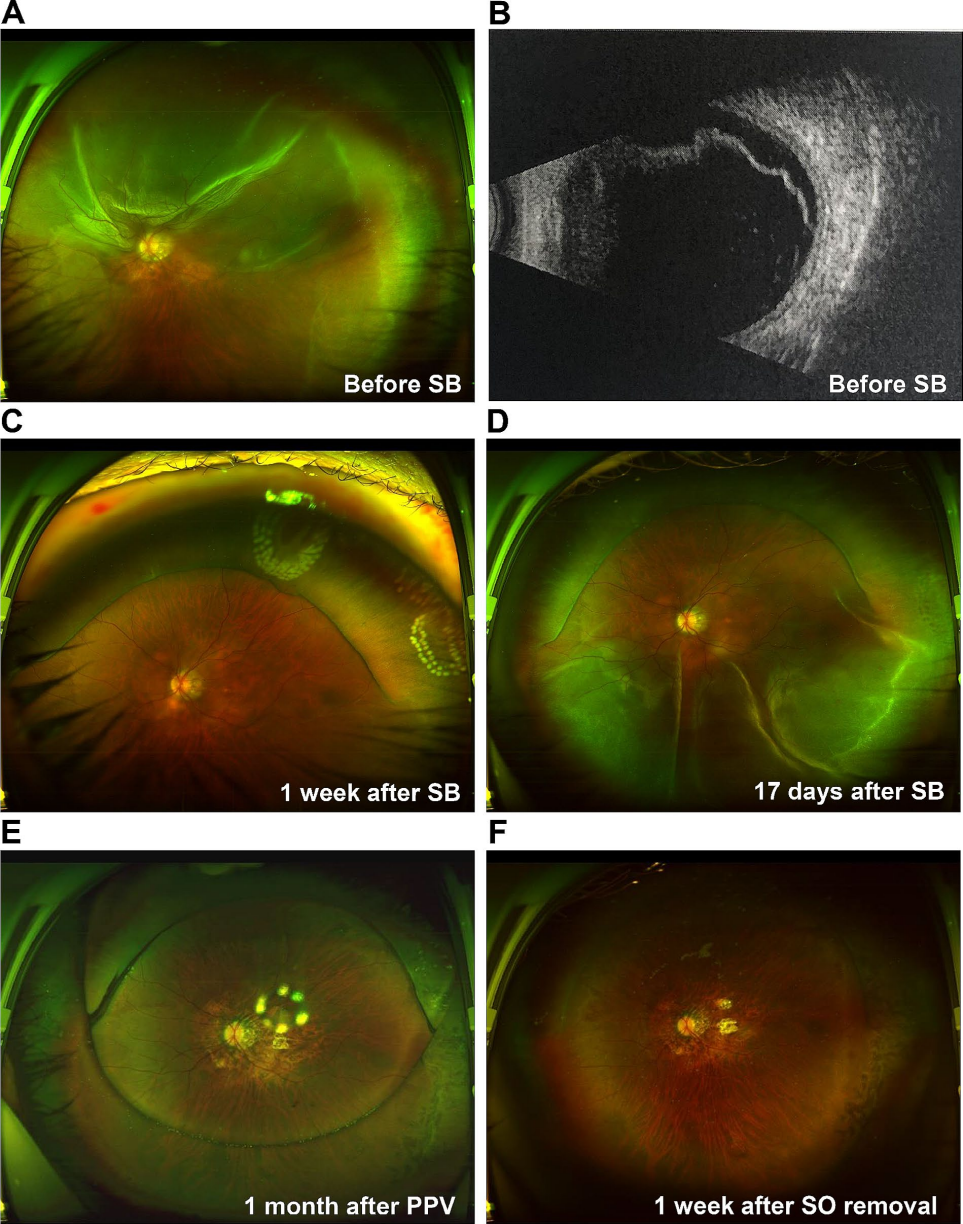
Ultra-widefield retinal imaging and B-scan images for Case 1 (**A**) Case 1 has macula-off superior RRD. (**B**) RRD image on the B-scan shows that the axial length is longer than normal. (**C**) Retinal reattachment 1 week after SB, with obvious scleral ridge and fresh laser spots. (**D**) Inferior retinal detachment 17 days after SB. (**E**) The retina is flat, and the vitreous cavity is filled with SO 1 month after PPV. (**F**) The retina is flat with obvious retinal atrophy 1 week after SO removal PPV: pars plana vitrectomy; RRD: rhegmatogenous retinal detachment; SB: scleral buckling; SO: silicon oil

**Fig. 2:**
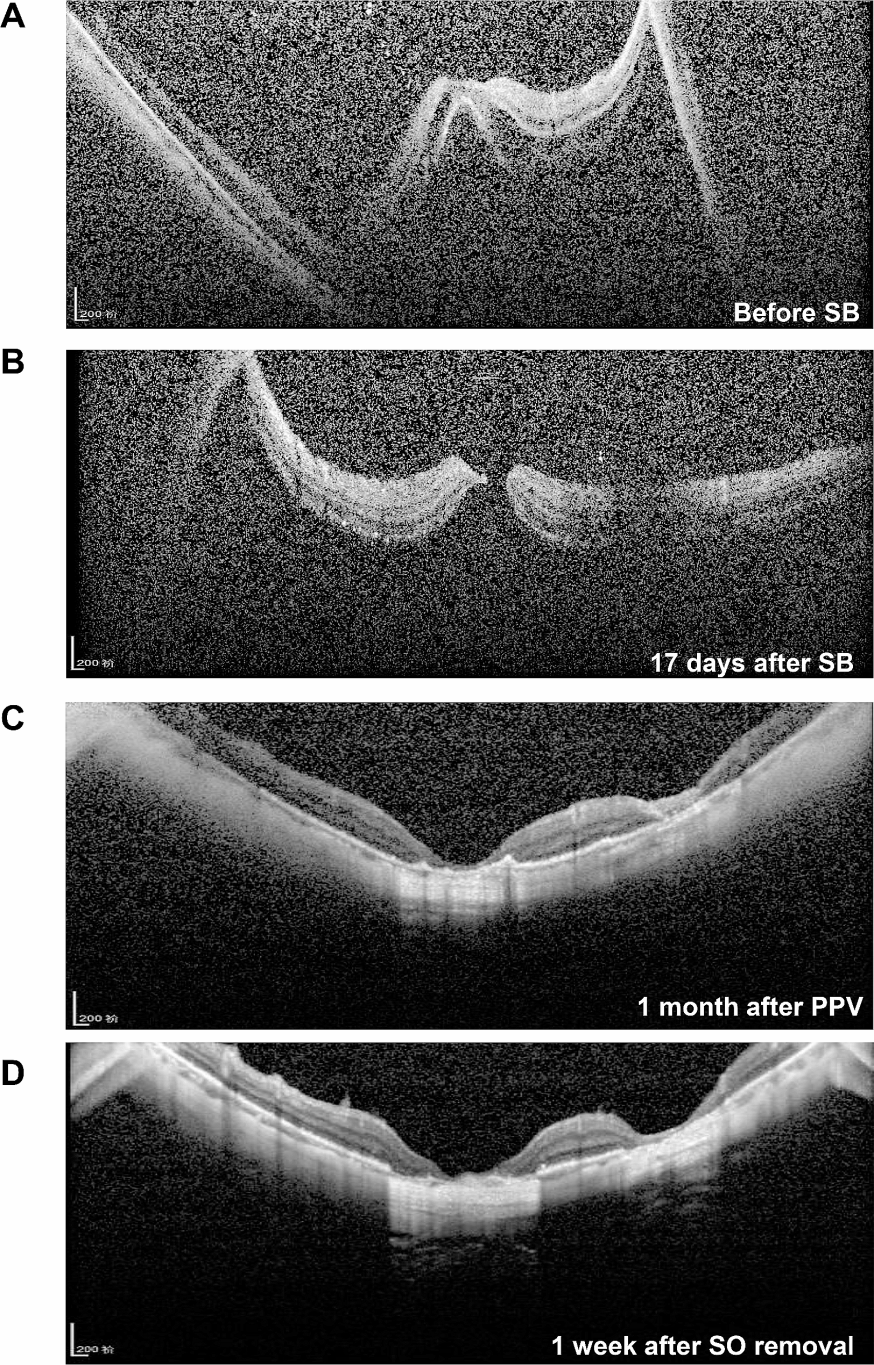
Optical coherence tomography images for Case 1 (**A**) Retinal detachment involving the macula is observed before SB. (**B**) Macular hole retinal detachment 17 days after SB. (**C**) Retinal reattachment and local retinal atrophy 1 month after pars plana vitrectomy. (**D**) The retina remains flat and retinal atrophy is obvious 1 week after SO removal PPV: pars plana vitrectomy; SB: scleral bucking; SO: silicon oil

The four patients underwent a series of imaging examinations, including B-scan ultrasonography, ultra-widefield retinal imaging, and OCT. Considering the many similarities among Cases 1–3, the imaging data of Case 1 are presented in Figs. 1 and 2. Prior to the SB procedure, Case 1 had macula-off superior RRD without MH (Fig. 1A and B, and 2A) and an axial length above normal (Fig. 1B). The patient developed retinal reattachment following SB, with obvious scleral ridge and fresh laser spots (Fig. 1C); however, 17 days after SB, Case 1 developed FTMH and MHRD (Figs. 1D and 2B). The patient subsequently underwent PPV, ILMP, and SOT, which closed the FTMH lesions (Figs. 1E and 2C), resulting in retinal reattachment. Finally, following silicon oil removal, the retina was still attached and the MH was closed with obvious local thinning and atrophy in the macular region (Figs. 1F and 2D).

**Fig. 3:**
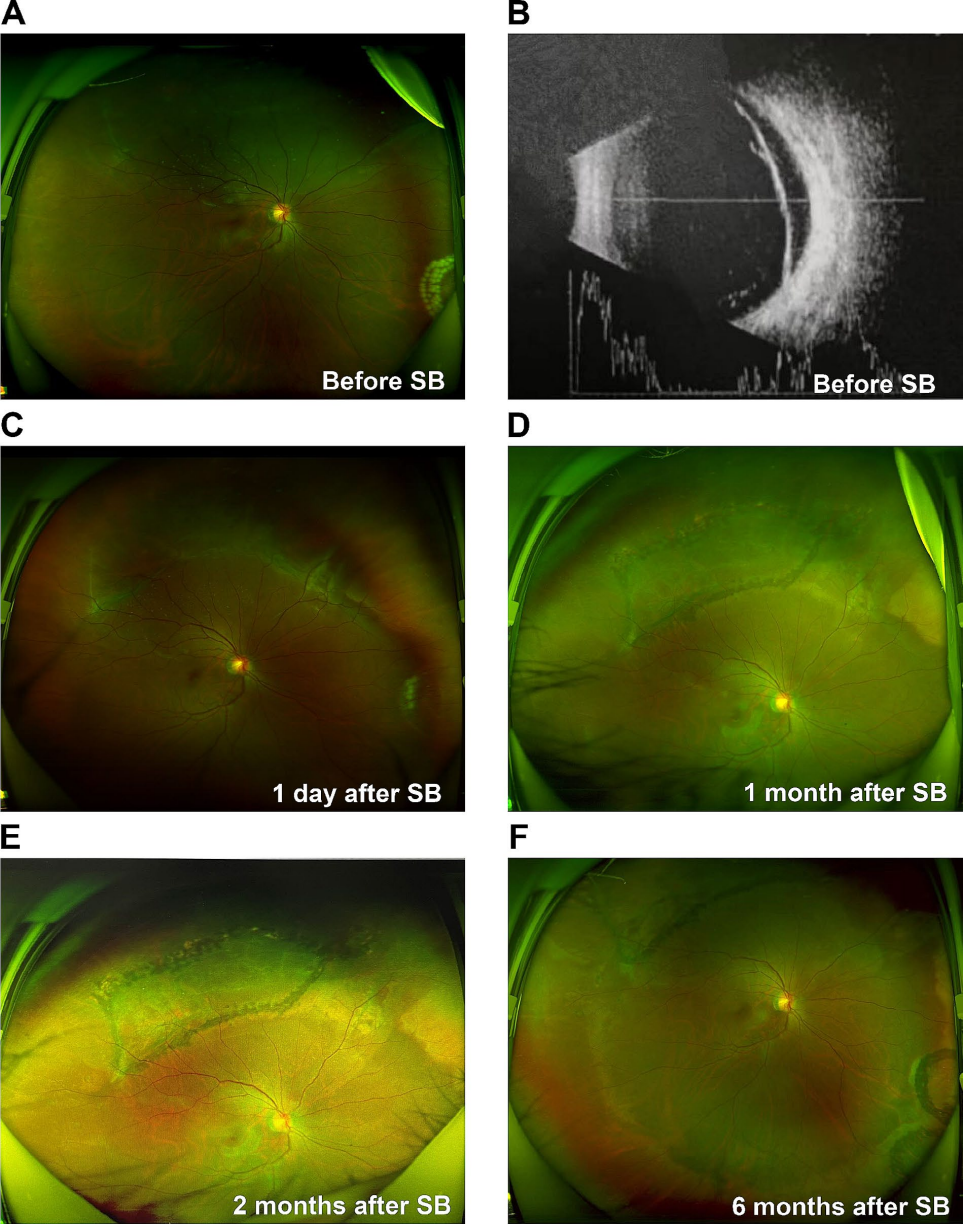
Ultra-widefield retinal imaging and B-scan images for Case 4 (**A**) Case 4 has superior macula-on RRD, and fresh laser spots can be seen in the inferior nasal retina. (**B**) RRD image on the B-scan shows that the axial length is normal. (**C**) Retinal reattachment with obvious scleral ridge and little subretinal fluid 1 day after SB. (**D**) The retina is flat with superior old laser spots 1 month after SB. (**E–F**) The retina is stable at 2 and 6 months following SB RRD: rhegmatogenous retinal detachment; SB: scleral buckling

**Fig. 4:**
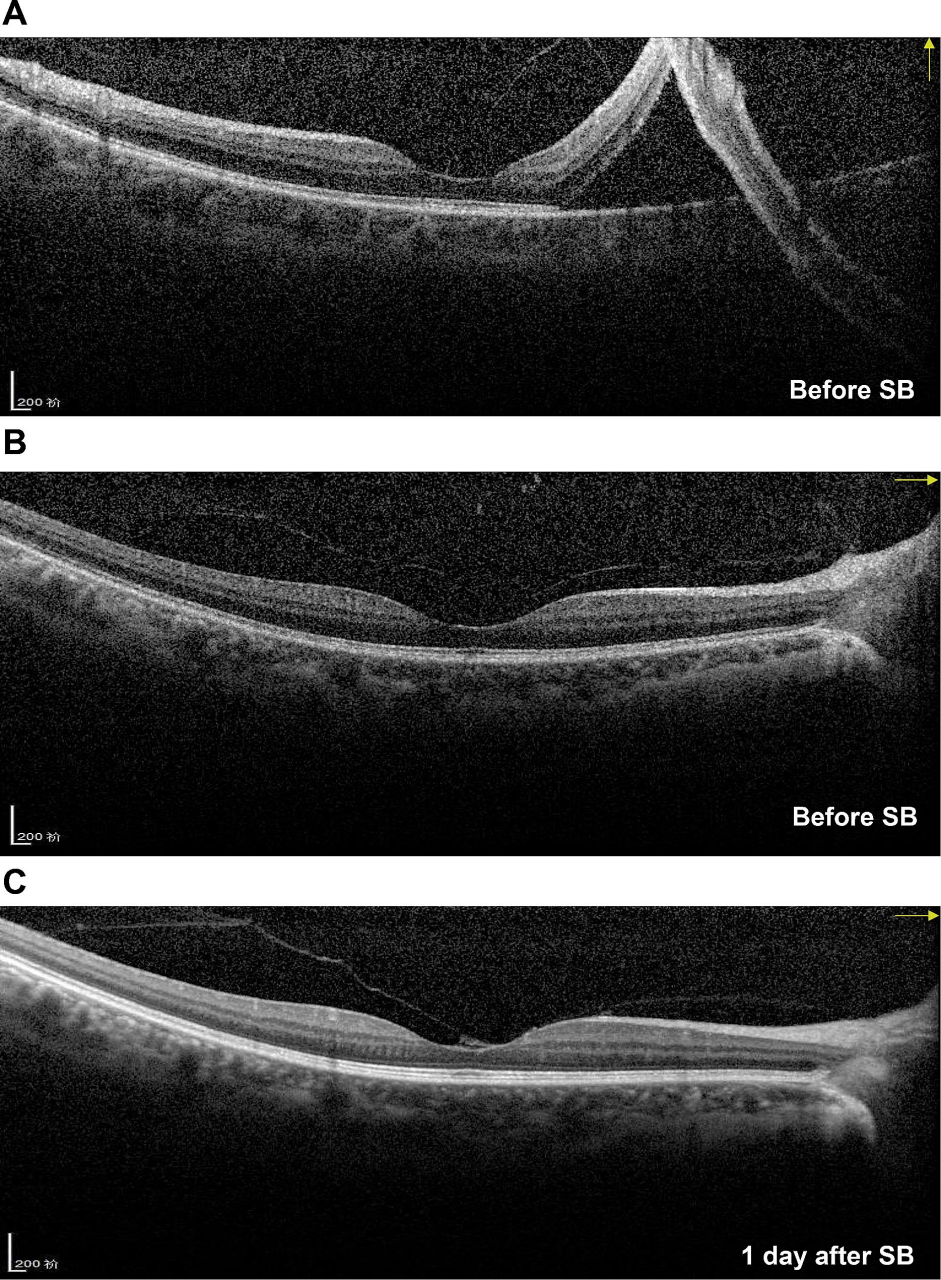
Optical coherence tomography images for Case 4 (**A**) The image shows macula-on superior rhegmatogenous retinal detachment with indistinct posterior vitreous cortex before SB. (**B**) The retina is flat in the horizontal direction, and the posterior vitreous cortex remains indistinct. (**C**) Vitreomacular adhesion can be observed 1 day after SB SB: scleral buckling

**Fig. 5:**
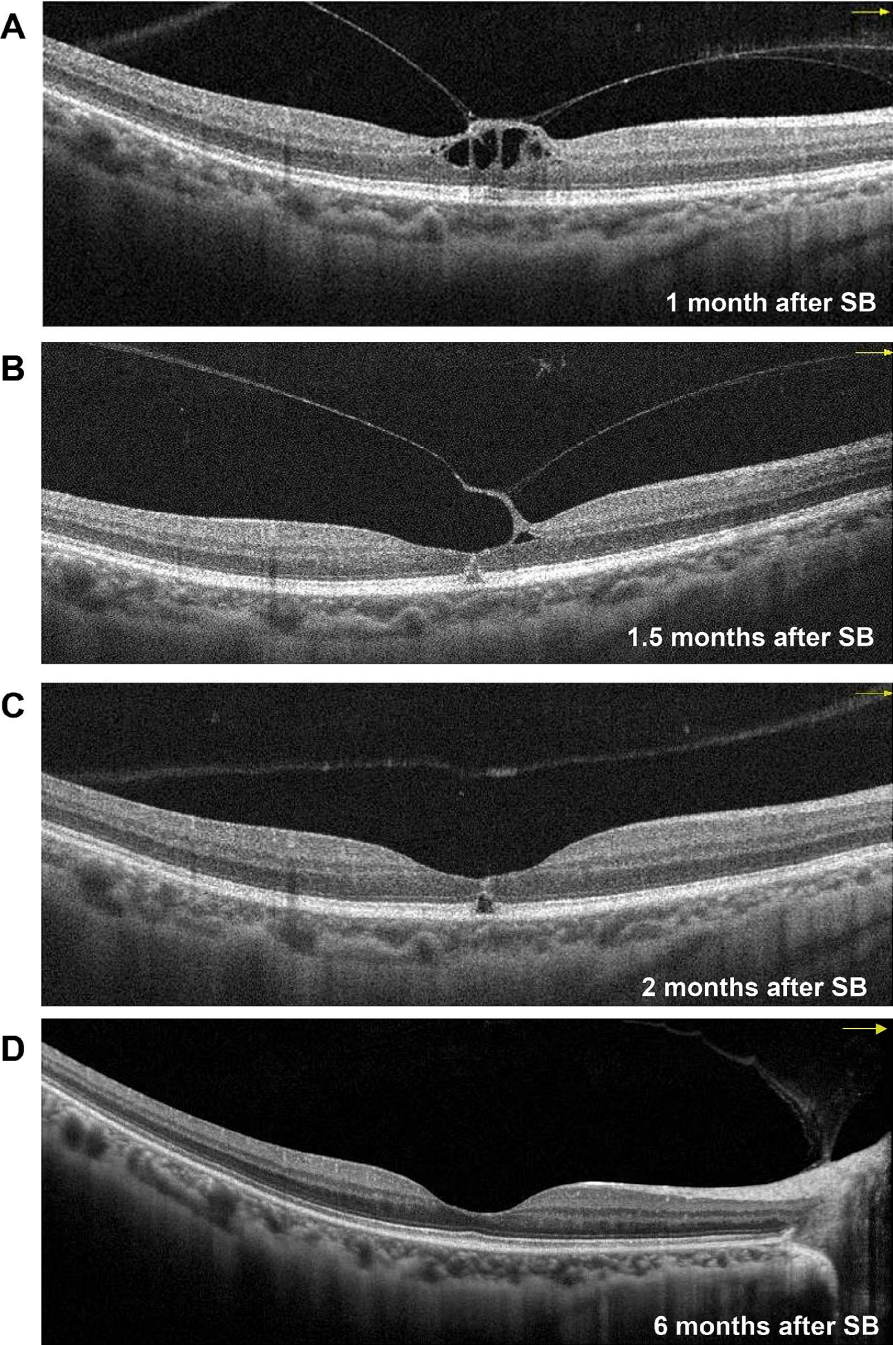
Follow-up optical coherence tomography images for Case 4 (**A**) Obvious VMT 1 month after SB, resulting in a cystic fovea. (**B**) VMT has developed, and the continuity of the retinal outer layer is destroyed 1.5 months after SB. (**C**) VMT is relieved, but a small defect in the retinal outer layer can be observed along with the formation of an obvious outer LMH 2 months after SB. (**D**) The outer LMH has recovered, leaving a point adhesion between the posterior vitreous cortex and the optic disc 6 months after SB LMH: lamellar macular hole; SB: scleral buckling; VMT: vitreomacular traction

The characteristics and outcomes of the disease of Case 4 were different from those of the other three cases; thus, the imaging data of Case 4 are presented in Figs. 3, 4 and 5. Before SB, Case 4 had macula-on superior RRD without MH (Fig. 3A and B, and 4A), with the detachment appearing very close to the fovea. The patient’s axial length was normal (Fig. 3B). Before the SB procedure, the posterior vitreous cortex (PVC) was indistinct in front of the retina (Fig. 4A and B). One day after SB, a vitreomacular adhesion was observed (Fig. 4C). One month after SB, vitreomacular traction (VMT) resulted in an obvious cystic fovea (Fig. 5A), and 1.5 months after SB, the VMT continued, destroying the continuity of the retinal layer (Fig. 5B). The VMT was relieved 2 months after SB; however, a small defect in the retinal outer layer and outer LMH was observed (Fig. 5C). Moreover, in the last follow up 6 months after SB, outer LMH recovery (Fig. 5D) as well as point adhesion between the PVC and the optic disc were observed. The patient’s retina remained flat postoperatively (Fig. 3C–F), and the visual acuity improved to 1.0.

## Discussion

RRD is a serious, vision-threatening condition, whose prevalence ranges from 6.3 to 17.9 per 10000 people and can result in blindness unless surgically treated [10]. At present, SB is one of the most common surgical methods for RRD. The development of postoperative MH following SB for RRD repair is a rare complication [11, 12]. Additionally, there are no relevant reports on the occurrence this complication in Chinese patients, and current reported cases only involve FTMH [11, 12]. In this study, we collected clinical data for 6 years from the largest ophthalmic centre in Northwest China. In addition, LMH following SB for RRD repair was first reported.

In our study, the prevalence of MH following SB for treating RRD was 0.83%, which is consistent with available data (0.54–0.86%) [13, 14]. Although the prevalence is low, the condition still deserves attention owing to its effect on central vision. Nevertheless, the prevalence was higher than those (0.20% [7] and 0.42% [15]) in other studies. However, data in those studies were based on patients undergoing RRD repair using treatment protocols other than just SB. The mean time of MH detection following SB was 26 days, with a range between 7 days and 23.5 months as reported in the literature [12, 13, 16]. In our study, MH occurred in one patient with macula-on RRD and three patients with macula-off RRD, with a prevalence of 0.32% and 1.85% in their respective populations. Additionally, MH was nearly six times more common in patients with macula-off RRD than in those with macula-on RRD but without statistical significance as demonstrated by the chi-squared test. Therefore, more cases should be included. Among the 41 cases reported in eight reports on MH following SB surgery, approximately 85% included patients with macula-off RRD [7, 11–17]. Thus, we should be more vigilant of this complication in patients with RRD involving the macula.

The potential mechanism of MH following SB for RRD repair could include the following: first, SB may increase the axial length, leading to aggravation of VMT; second, SB combined with an encircling band could affect ocular circulation; third, SB does not resolve the inflammation in the vitreous cavity.

In the present study, PVD was observed in Cases 1–3 as well as in other studies; however, VMT is still considered the main cause of MH [14, 15]. PVD is defined as the separation of the posterior cortical vitreous from the internal limiting membrane [18]. It usually begins as a shallow, localised separation of the vitreous from the perifoveal retina and progresses slowly until its completion at the time of vitreopapillary separation [18]. Therefore, Weiss ring lesions have been often used as a criterion for evaluating PVD, but they only represent the detachment between the posterior hyaloid and optic disc and do not indicate the absence of VMT [12]. If PVD and VMT are to be evaluated objectively, OCT may be the best choice [19]. However, the status of the detached retina interferes with the reliable evaluation of PVD or VMT in an OCT image (especially macula-off RRD, as shown in Cases 1–3), and there is no published report on the evaluation of PVD using OCT in patients with RRD [20]. Cases 1–3 had high myopia; in addition, anomalous PVD may have occurred in these patients due to accelerated vitreous liquefaction before adequate weakening of the vitreoretinal adhesion [21]. Furthermore, preoperative fundus observation (whether with ophthalmoscopy or OCT) would be affected owing to pigment particles and inflammation in the vitreous cavity, which may affect the PVD assessment. As a result, even if Cases 1–3 had PVD, we could not rule out the absence of preoperative VMT, which is a risk factor for postoperative MH. However, another explanation could be that pre-existing PVD damages the inner retina, such as subtle defects or breaks in the internal limiting membrane [13, 22]. Importantly, the formation of outer LMH observed with OCT in our study confirmed the role of VMT in MH formation following SB, which has not been reported or confirmed using OCT previously [13]. How to accurately assess preoperative PVD in patients with RRD (especially involving the macula and in those with high myopia) is a challenge for both technologists and surgeons.

Furthermore, whether PVD affects the choice of surgical method in patients with RRD is worthy of discussion. The development of RRD typically involves three factors: one or more full-thickness breaks in the retina, PVD, and passage of fluid from the vitreous cavity through the retinal breaks into the potential subretinal space [23]. However, RRD without PVD can also develop [24]. Research has shown that simple SB is suitable for RRD with less liquefied vitreous humour and without PVD, as the formed vitreous can act as a ‘bio-tamponade’ to block the passage of fluid, and traction to the retinal break associated with PVD and vitreous liquefaction may prevent break closure [20]. However, SB can also be used in patients with RRD and PVD and may need to be combined with other external procedures, such as drainage of the SRF or injection of gas [20]. There are no unified formal guidelines for selecting the optimal surgical procedure for the repair of RRD [23]. The surgical treatment of RRD remains a highly individual matter that is influenced by the preoperative findings, patient characteristics, available tools for surgery, and experience and ability of the operating surgeon [25]. A multicentre, prospective clinical trial showed a benefit of SB in phakic eyes with respect to BCVA improvement compared with PPV [25]. Therefore, in clinical practice, it is generally believed that SB is an ideal procedure for RRD in young and phakic eyes [26], such as the patients in this study. It is also important to note that the risk of postoperative MH is a challenge if patients without PVD are selected for SB.

Considering the ocular circulation, retinal ischaemia in the central fovea owing to retinal separation from the choroidal vascular supply plays an important role [14]. In the present study, the range of retinal detachment in a patient with outer LMH postoperatively did not involve the macula, albeit it was close to the macula, which could still affect macular circulation. Moreover, the pressure from SB and the encircling band could affect the blood supply of the eyeball. Lastly, SB did not disturb the intraocular microenvironment, but it did not resolve the intraocular inflammation, which could exacerbate the macular disorder.

There are other viewpoints that explain the pathogenesis of MH following SB, such as epiretinal membrane (ERM) and cystoid macular oedema (CME) [12]. In our study, we did not observe ERM or CME in any of the cases. The prevalence of ERM is positively correlated with age [27]. Half of the participants in the study by Garcia et al. [12] had ERM, and the average age of the patients was 54 years, whereas that of patients in the present study was 43.5 years. Considering CME, we reviewed the original literature demonstrating that CME usually appeared after PPV or in patients with diabetic retinopathy. In the present study, except for Cases 1–3 with high myopia, there were no CME-related ocular factors as mentioned above. Furthermore, discussions on the relationship between high myopia and postoperative MH are scant. In the previous eight studies, only 10% of the patients had high myopia, and some studies did not mention the refractive status of the patients [7, 11–17]. However, 75% of the patients in our study had high myopia and postoperative FTMH. Nearly all previous reports were from Europe and America; however, myopia is a common disease in East and Southeast Asia [28]. An unhealthy vitreomacular interface, thin retina and choroid, and poor tolerance to decreased circulation make patients with high myopia more susceptible to MH. Thus, we should pay more attention to these patients.

All three patients with RD in this study had high myopia. The most commonly used measurement method, such as optical biometry or A-scan ultrasound, may not provide accurate axial length measurements in patients with retinal detachment as the patients’ fixation is hampered by the detached retina or macula [29]. In this study, the B-scan mode of Quantel was used to measure the axial length. Ultrasonic measurement of axial length in patients with retinal detachment may be fallacious; thus, A-scan was introduced to the module to combine A- and B-scan, which was similar to that employed in Mohsen’s study. Moreover, this study showed that A- combined with B-scan is a good choice for patients with retinal detachment [29]. Nevertheless, accurate measurement of the axial length in these patients is challenging.

In our study, all patients with FTMH underwent reoperation owing to MHRD, and the FTMH closed after the operation, thus enabling retinal reattachment. Cases of FTMH that developed after SB and closed spontaneously without surgical treatment have also been reported [11]. However, in previous studies, cases of FTMH closure without reoperation accounted for a minority of the total observed cases. Additionally, the closure rate of MH also varied among studies [7, 15]. In the present study, postoperative LMH following SB was reported for the first time, and we were able to conduct a close follow up without surgical treatment owing to the patient’s compliance. Therefore, based on different situations, reasonable treatment judgments should be made.

Although our study summarised clinical data of 6 years from the largest ophthalmic centre in Northwest China, the present study still has certain limitations. First, it was a retrospective study with a small sample size. Second, preoperative OCT was performed in the patients; however, complex and severe RD may affect the OCT results, which is a limitation of this study. Therefore, adequate fundus examination should be performed before surgery to minimise the omission of MH. Moreover, the prevalence of MH following SB could have been underestimated since not all 483 patients underwent regular OCT after surgery in our study. Additionally, some patients were not followed up regularly, possibly owing to the impact of the coronavirus disease pandemic. Therefore, larger studies, preferably from multiple centres, with more data concerning MH following SB for the treatment of RRD, as well as deeper research and greater effort, are warranted.

## Conclusions

Although MH secondary to SB for RRD repair is a relatively rare postoperative complication, it still deserves attention. Furthermore, it is important to note that both FTMH and LMH may occur after SB for the treatment of RRD. Patients with high myopia combined with macula-off RRD may be more susceptible to FTMH, which may cause retinal re-detachment. Therefore, we should raise awareness of MH following SB for RRD repair.

## Data Availability

The data used in this study are included in the article and are available upon reasonable request from the corresponding author.

## Abbreviations

BCVA: Best corrected visual acuity
CME: Cystoid macular oedema
ERM: Epiretinal membrane
FTMH: Full-thickness macular hole
ILMP: Internal limiting membrane peeling
IOP: Intraocular pressure
LMH: Lamellar macular hole
MH: Macular hole
MHRD: Macular hole retinal detachment
OCT: Optical coherence tomography
PPV: Pars plana vitrectomy
PVC: Posterior vitreous cortex
PVD: Posterior vitreous detachment
RRD: Rhegmatogenous retinal detachment
SB: Scleral buckling
SOT: Silicon oil tamponade
SRF: Subretinal fluid
VMT: Vitreomacular traction
